# The WHO safe childbirth checklist after 5 years: future directions for improving outcomes

**DOI:** 10.1016/S2214-109X(21)00556-8

**Published:** 2022-02-15

**Authors:** Rose L Molina, Lauren Bobanski, Neelam Dhingra-Kumar, Allisyn C Moran, Ayda Taha, Somesh Kumar, Katherine E A Semrau

**Affiliations:** a Ariadne Labs, Brigham and Women's Hospital, Harvard T.H. Chan School of Public Health, Boston, MA 02215, USA; b Department of Obstetrics and Gynecology, Beth Israel Deaconess Medical Center, Boston, MA, USA; c Harvard Medical School, 25 Shattuck St, Boston, MA 02115, USA; d WHO Patient Safety Flagship, Department of Integrated Health Services, WHO, Geneva, Switzerland; e Department of Maternal, Newborn, Child and Adolescent Health and Ageing, WHO, Geneva, Switzerland; f Jhpiego, New Delhi, India; g Department of Medicine, Brigham and Women's Hospital, Boston, MA USA

There is growing global momentum to improve quality of childbirth care and optimise experience and outcomes. In September 2021, World Patient Safety Day was dedicated to safe maternal and newborn care. The WHO Safe Childbirth Checklist (SCC),^1^ designed out of the patient safety movement, addresses the major causes of morbidity and mortality surrounding childbirth and was publicly released in 2015. The SCC consolidates essential birth practices that should be done for every woman and infant. Although the SCC was designed as a job aide for frontline professionals, the SCC alone is not what drives safety and quality. Rather, the SCC has been most effective when implemented as part of larger quality improvement initiatives that enhance the enabling environment—policies and practices that enable success—for childbirth care.

The BetterBirth trial,^2^ the largest trial of the SCC to date, showed that SCC implementation with an 8-month coaching programme and continuous data feedback led to significant improvements in adherence to evidence-based practices in primary-level facilities in Uttar Pradesh, India, but did not lead to a reduction in maternal or perinatal morbidity and mortality. This disconnect between behaviour change and outcomes was puzzling. Outcomes might not have improved because implementation occurred in primary-level facilities with baseline poor quality of care and essentially no caesarean section delivery capacity. A post-hoc analysis of observed deliveries in the BetterBirth trial showed significantly reduced odds of perinatal and early neonatal mortality with each additional checklist practice performed.^3^ This finding suggests that there might need to be a high threshold of adherence to a set of checklist items to have an impact on outcomes, requiring quality improvement infrastructure and an enabling environment.

Evidence highlights the impact of the SCC on perinatal outcomes when implemented as part of broader quality improvement interventions. In Rajasthan, India, a quasi-experimental study showed that stillbirths and very early neonatal deaths dropped after implementing the SCC with supportive coaching and provision of medications and equipment in secondary-level facilities.^4^ A cluster-randomised facility-based trial in Kenya and Uganda showed reductions in fresh stillbirth and neonatal mortality among preterm and low-birthweight neonates when an adapted SCC was implemented with data strengthening, team training, and quality improvement collaboratives in rural and peri-urban facilities.^5^ Yet maternal morbidity and mortality remain challenging to improve. Identifying the key complementary initiatives to improve maternal outcomes is a critical next step.

Implementers around the world have taken up the SCC and adapted, implemented, and evaluated it because of its value in improving safety and quality of care.^6^ Several studies have replicated increased adherence to practices in the SCC using various adaptations and implementation approaches.^7^, ^8^, ^9^, ^10^ Although the uptake of the SCC is noteworthy, there remains an unfinished agenda to identify adaptations—both clinical and operational—to ensure key practices are done for every birthing person, every time.^6^ The evidence base and context for a variety of childbirth practices will probably change over time; therefore the SCC should incorporate new evidence and guidelines. For example, there might be an emerging need to include COVID-19-specific tasks. Additionally, the WHO Labour Care Guide and forthcoming guidelines on postnatal care of small and sick neonates should be included in future adaptations.

Regarding optimal implementation pathways for the SCC, much remains unknown. Different strategies (such as coaching and incentives) might be needed to motivate behaviour change for particularly difficult practices. The content, frequency, and duration of coaching to sustain behaviour change requires investigation in different settings. Facility preparedness requires appropriate infrastructure, supplies and equipment, qualified personnel, transportation and referral systems, and communication lines across all levels of care. Additionally, key enabling environmental factors to facilitate adherence to SCC practices—technical team training, quality improvement infrastructure, and leadership support—are crucial to success.

The SCC has been implemented in various settings with constrained resources, precluding evaluations that require substantial data infrastructure. Standard definitions of quality metrics are needed to harmonise evaluation efforts. To date, there are no agreed upon scalable metrics for patient experience and few metrics for facility and health system readiness. Increased investment is needed to build the data infrastructure for real-world implementation studies and avoid duplication of uncoordinated data collection.

A growing body of evidence shows the impact of the SCC on adherence to evidence-based practices, and its potential effect on perinatal morbidity and mortality when implemented within broader quality improvement initiatives. The SCC remains an important patient safety tool for high-reliability systems. Yet the optimal adaptations and implementation pathways to impact outcomes in a given context remain elusive. With strong signals of effectiveness emerging from early adopters, there is an opportunity to set the agenda for the next 5 years for the SCC (appendix). Now is the time to identify the optimal complementary intervention packages and contexts for the SCC to improve maternal and newborn safety, quality, and outcomes.

We declare no competing interests.
