# Supplementary material for: The WHO safe childbirth checklist after 5 years: future directions for improving outcomes
Source: Lancet Glob Health. 2022 Feb 15;10(3):e324–5. doi: 10.1016/S2214-109X(21)00556-8 (PMC8864300; doi:10.1016/S2214-109X(21)00556-8)
Supplement: Supplementary appendix [file mmc1.pdf]

THE LANCET
Global Health

Supplementary appendix

This appendix formed part of the original submission. We post it as supplied by the authors.

Supplement to: Molina RL, Bobanski L, Dhingra-Kumar N, et al. The WHO safe childbirth checklist after 5 years: future directions for improving outcomes.
Lancet Glob Health 2022; **10:** e324–25.

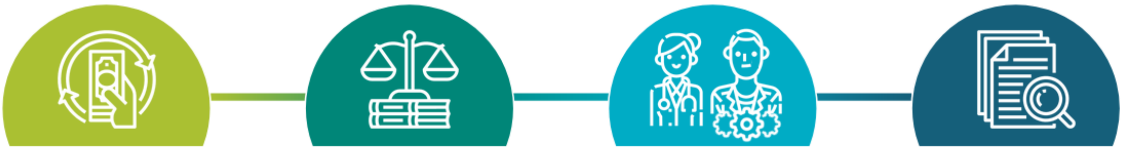

DONORS

- Invest in interventions & enabling environments
- Support data harmonization across funded projects

MINISTRIES OF HEALTH & POLICYMAKERS

- Identify gaps in enabling environments & create supportive policies with appropriate funding

CLINICIANS & IMPLEMENTERS

- Continue to take up SCC as part of QI initiatives
- Share lessons learned about adaptation & implementation through a Community of Practice and link to other existing networks
- Incorporate new evidence-based guidelines into SCC

RESEARCHERS

- Apply implementation science to understand intervention & implementation successes & challenges
- Address complexity around childbirth through complementary bundles
- Harmonize quantitative & qualitative metrics
